# Fluctuation X-ray scattering from nanorods in solution reveals weak temperature-dependent orientational ordering

**DOI:** 10.1107/S2052252519005499

**Published:** 2019-05-22

**Authors:** Ruslan P. Kurta, Lutz Wiegart, Andrei Fluerasu, Anders Madsen

**Affiliations:** a European XFEL, Holzkoppel 4, 22869 Schenefeld, Germany; b Brookhaven National Laboratory, Photon Sciences Directorate, Upton, NY 11973, USA

**Keywords:** nanoscience, small-angle X-ray scattering, SAXS, correlated fluctuations, X-ray free-electron lasers, XFELs

## Abstract

Higher-order statistical analysis of X-ray scattering from dilute solutions of polydisperse goethite nanorods was performed and revealed structural information which is inaccessible by conventional small-angle scattering. For instance, a pronounced temperature dependence of the correlated scattering from suspension was observed. The higher-order scattering terms deviate from those expected for a perfectly isotropic distribution of particle orientations, demonstrating that the method can reveal faint orientational order in apparently disordered systems. The observation of correlated scattering from polydisperse particle solutions is also encouraging for future free-electron laser experiments aimed at extracting high-resolution structural information from systems with low particle heterogeneity.

## Abstract 2

Angular cross-correlation analysis of X-ray scattering from solutions of nanoparticles demonstrates high sensitivity to orientational particle inhomogeneities.

## 1. Introduction   

Non-crystalline materials, such as glasses, liquids and solutions, can accommodate various structural features which are intrinsically forbidden in systems with translational symmetry. Traditionally, X-ray scattering studies of disordered matter rely on small-angle X-ray scattering (SAXS) and pair-distribution function (PDF) analysis (Als-Nielsen & McMorrow, 2011 ▸; Warren, 1990 ▸). These widely used approaches, however, provide only limited structural information and are usually insufficient for the unambiguous derivation of the 3D structure that is crucial for a complete understanding of a material’s properties. Exploring higher-order statistics of the scattered intensity can provide additional information about disordered systems beyond that accessible by conventional analyses. It has been suggested that, by calculating angular cross-correlation functions (CCFs) of the scattered intensity images, it may be possible to extract higher-order scattering terms preserved in the measured intensity fluctuations beyond the isotropic averages (Kam, 1977 ▸). By performing so-called fluctuation X-ray scattering (FXS) experiments, one could, for example, facilitate biological structure determination from solution scattering (Kam, 1977 ▸; Kam *et al.*, 1981 ▸) or detect local orientational order and hidden symmetries in amorphous materials (Clark *et al.*, 1983 ▸; Ackerson *et al.*, 1985 ▸).

Practical implementation of these techniques became possible only recently, mostly due to advances in X-ray instrumentation (Kurta *et al.*, 2016 ▸). X-ray cross-correlation analysis (XCCA) based on CCFs allowed details to be revealed of the structural arrangements in partially ordered soft-matter systems such as colloids (Wochner *et al.*, 2009 ▸; Lehmkühler *et al.*, 2014 ▸; Schroer *et al.*, 2015 ▸, 2016 ▸), liquid crystals (Kurta *et al.*, 2013*c* ▸; Zaluzhnyy *et al.*, 2015 ▸, 2017*b* ▸, 2018 ▸), polymer blends (Kurta *et al.*, 2015 ▸) and mesostructures (Zaluzhnyy *et al.*, 2017*a* ▸; Mancini *et al.*, 2016 ▸; Lhermitte *et al.*, 2017 ▸). Correlations have also been explored in electron scattering (Treacy *et al.*, 2005 ▸, 2007 ▸; Treacy & Borisenko, 2012 ▸; Liu *et al.*, 2013*a* ▸, 2015 ▸) where the requirement for a small scattering volume can be conveniently achieved to observe the intensity fluctuations associated with atomic scale structures (Clark *et al.*, 1983 ▸). The revival of the field by new experimental X-ray capabilities has been accompanied by novel developments in the theory of correlated scattering and advanced data analysis (Altarelli *et al.*, 2010 ▸; Kirian *et al.*, 2011 ▸; Malmerberg *et al.*, 2015 ▸; Liu *et al.*, 2016 ▸; Martin, 2017 ▸).

With the emergence of X-ray free-electron lasers (XFELs), the FXS approach has been extensively revised for biological structure determination from solution scattering (Saldin *et al.*, 2009 ▸, 2011 ▸; Poon & Saldin, 2011 ▸; Kurta *et al.*, 2012 ▸, 2013*b* ▸; Liu *et al.*, 2012 ▸; Chen *et al.*, 2013 ▸; Malmerberg *et al.*, 2015 ▸; Kurta, 2016 ▸). Experimental demonstrations for disordered ensembles of various engineered nanostructures like nano-rice (Liu *et al.*, 2013*b* ▸), polymer dumb-bells (Chen *et al.*, 2012 ▸; Starodub *et al.*, 2012 ▸) and three-bladed nano-propellers (Pedrini *et al.*, 2013 ▸) indicated the general feasibility of the FXS approach. In particular, FXS measurements on crystalline nanoparticles in solution demonstrated the possibility of measuring atomic scale correlated scattering (Mendez *et al.*, 2014 ▸, 2016 ▸). Recently, the FXS approach has successfully been applied to data taken at the Linac Coherent Light Source (LCLS) in the USA for biological structure determination. In combination with a novel iterative phasing algorithm (*MTIP*; Donatelli *et al.*, 2015 ▸), FXS allowed the reconstruction of aerosolized single virus particles (Kurta *et al.*, 2017 ▸) and multiple virus particles in solution (Pande *et al.*, 2018 ▸) with nanometre precision.

In studies of FXS from solution, a uniform distribution of particle orientations is often assumed since it is a necessary requirement for a successful 3D reconstruction. The question is whether such a requirement is strictly fulfilled in real experiments and how it affects the resolution. Clearly, interparticle interaction may be responsible for the appearance of orientational particle correlations in concentrated solutions. In a generic SAXS experiment, the thermodynamic argument can be neglected when using dilute particle solutions, where vanishingly small particle–particle interactions result in a structure factor value close to unity. The effect of orientational order, however, has not been explored in FXS experiments, where the high sensitivity to orientational inhomogeneities may lead to the manifestation of subtle thermodynamic effects in the FXS data. On the other hand, particle motion during X-ray exposure, particularly rotational diffusion for elongated particles, can blur the contrast of the FXS data. In this work we investigate how the orientational distribution and rotational diffusion of particles in solution affect experimental FXS data. Measurements were performed on aqueous suspensions of polydisperse goethite nanorods at different volume fractions and temperatures. Our results show a pronounced temperature dependence of the correlated scattering which, to a great extent, can be associated with orientational particle correlations.

The analysis reveals that the higher-order scattering terms have larger values than expected for an isotropic distribution of particle orientations, indicative of weak orientational (nematic) ordering. This demonstrates that FXS can also be used as a high-sensitivity probe of orientational order in apparently disordered systems.

## 2. Theoretical background   

We consider X-ray scattering from a dilute polydisperse mixture of *N* particles. The ensemble-averaged SAXS intensity for such a system can be specified as (Als-Nielsen & McMorrow, 2011 ▸)
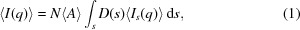
where *A* is an experimental normalization factor (see Appendix *A* [App appa]), *I*_*s*_(*q*) is the scattered intensity at a momentum transfer of magnitude *q* for a particle of size *s* and *D*(*s*) is the normalized particle size distribution function, so 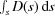 = 1. The integration is performed over all particle sizes *s*, and 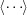 denotes statistical averaging. The SAXS intensity, equation (1), commonly used to characterize polydisperse systems, can be interpreted as a zeroth-order term in the context of our work.

Here we introduce higher-order scattering terms for a dilute polydisperse system of particles as (see Appendix *A* [App appa] for more details)
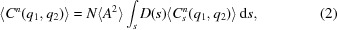
where 〈*C* ^*n*^(*q*_1_, *q*_2_)〉 is the ensemble-averaged *n*th order Fourier component (FC) of the angular cross-correlation function (CCF) *C*(*q*_1_, *q*_2_, Δ) [see *e.g.* Kam (1977 ▸) and Kurta *et al.* (2016 ▸)], defined here for a polydisperse system of particles, and 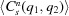 are the ensemble-averaged FCs defined for a specific particle size *s*. In equation (2) it is assumed that the particle orientations are uniformly distributed and 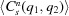 is in fact a single-particle quantity.

Equation (2) defines higher-order scattering terms which are unavoidably lost in the isotropic SAXS intensity equation (1) and which we seek to extract here. For practical applications, the FCs 〈*C* ^*n*^(*q*_1_, *q*_2_)〉 can be experimentally approximated by the so-called difference FCs 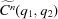 (see Appendix *B* [App appb]) which help in reducing the effect of various errors in the experimental data analysis (Kurta *et al.*, 2017 ▸).

## 3. Experiment   

The SAXS experiment was performed on beamline ID10 at the European Synchrotron Radiation Facility (ESRF, France) with 10 keV photon energy at about 1.3% bandwidth (pink beam). Using a double-mirror system and a set of slits, the X-ray beam was focused and collimated to a size of about 20 × 20 µm with about 10^13^ photons s^−1^ hitting the sample. The scattered intensity was recorded by a Maxipix detector (consisting of 256 × 256 square pixels, 55 µm in size) situated 515 mm downstream from the sample. A 2 mm round beamstop of Pb was placed in front of the detector to protect it from the direct beam transmitted through the sample.

The image acquisition time was 1 ms to minimize rotational motion of the nanoparticles during exposure, and a fast shutter before the sample protected it from the X-ray beam during the 0.1 s of waiting time between successive exposures. Scattering measurements were performed on dilute aqueous solutions (80 wt% propane-1,3-diol in water) of goethite (α-FeOOH) nanorods contained in glass capillaries of diameter 0.7 mm. About 10^4^ diffraction patterns were acquired for the correlation analysis to accumulate sufficient statistics for each volume fraction of goethite particles (φ_g_ = 0.05% and 0.5%) at the different temperatures.

The powerful X-ray beam is potentially able to damage the sample, for instance resulting in gas bubbles that would lead to very strong and unwanted stray scattering. Hence, a procedure was established where every spot of the capillary only received an exposure of 10–20 ms, after which a new spot was illuminated. The waiting time between exposures ensures that diffusion creates a new spatial arrangement of nanoparticles inside the scattering volume, which is important for correct ensemble averaging.

The structure and dynamics of goethite solutes have previously been studied by X-ray scattering (Lemaire *et al.*, 2004 ▸; Poulos *et al.*, 2010 ▸). For the suspensions used here it has been established in structure factor studies that there is no orientational (nematic) ordering below about 4% volume concentration of particles. The exposure time of 1 ms was chosen as a compromise between the need for a strong scattering signal and the requirement that the particles remain in quasi-fixed positions during exposure. The latter can be ensured by cooling the sample, since the solvent (80 wt% propane-1,3-diol in water) increases in viscosity at low *T* and hence slows down the rotational diffusion over 1 ms, from ∼10° at room temperature (297 K) to ∼1° at 229 K and ∼0.1° at 209 K.

## 4. Results and discussion   

**Figure 1:**
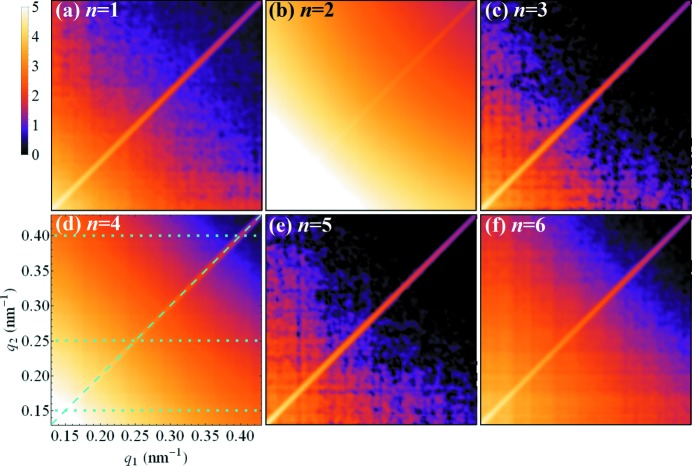
Amplitudes (log scale, arbitrary units) of the difference FCs 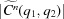 for *n* = 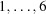, calculated from X-ray scattering images for the sample with a volume fraction φ_g_ = 0.05% of goethite nanorods at *T* = 229 K. The colour map and the axes specified in panel (*d*) are the same for all maps. The dashed line at *q*_1_ = *q*_2_ = *q*, and the dotted lines at *q*_2_ = 0.15, 0.25 and 0.40 nm^−1^ in panel (*d*) indicate sections through the 2D maps (maps for *n* > 6 are not shown here). These sections are shown in Fig. 2 ▸.

**Figure 2:**
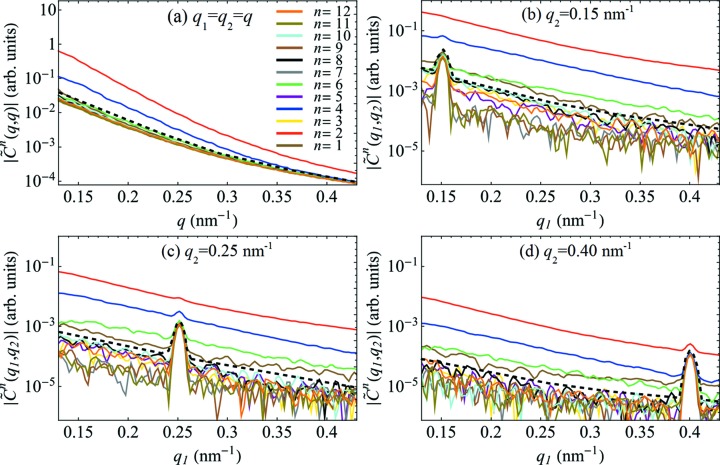
Amplitudes (log scale) of the difference FCs 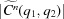 for *n* = 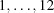, determined at (*a*) *q*_1_ = *q*_2_ = *q*, (*b*) *q*_2_ = 0.15 nm^−1^, (*c*) *q*_2_ = 0.25 nm^−1^ and (*d*) *q*_2_ = 0.40 nm^−1^ as a function of *q*_1_ (see Fig. 1 ▸). Three dominant FCs of the orders *n* = 2 (red), *n* = 4 (blue), and *n* = 6 (green) clearly stand out from the background level [proximately indicated with dashed lines] formed by the degenerate FCs of other orders.

SAXS images were corrected for background scattering and normalized by the average intensity per pixel. Saturated pixels, dead pixels and pixels shadowed by the beamstop were masked in the analysis, and a flat-field correction was applied to the detector. For the correlation analysis we employed the difference spectra 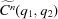 (see Appendix *B* [App appb]), calculated in the range of scattering vectors from *q* = 0.13 to 0.43 nm^−1^. Fig. 1 ▸ shows a set of 2D correlation maps illustrating the amplitudes of six FCs 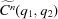, *n* = 1–6, determined at φ_g_ = 0.05% and *T* = 229 K. Dotted and dashed lines in Fig. 1 ▸(*d*) define four different sections through the 2D maps. These sections are shown in detail in Fig. 2 ▸ for *n* = 1–12.

One can see from Figs. 1 ▸ and 2 ▸ that within the analysed portion of reciprocal space, a dominant contribution to the difference spectrum 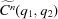 originates from FCs of low even orders, *i.e.* *n* = 2, 4 and 6. Apart from a slightly increased value of the *n* = 1 FC, most probably due to imperfect centring of the SAXS patterns and/or absorption effects, all other FCs have vanishing values.

**Figure 3:**
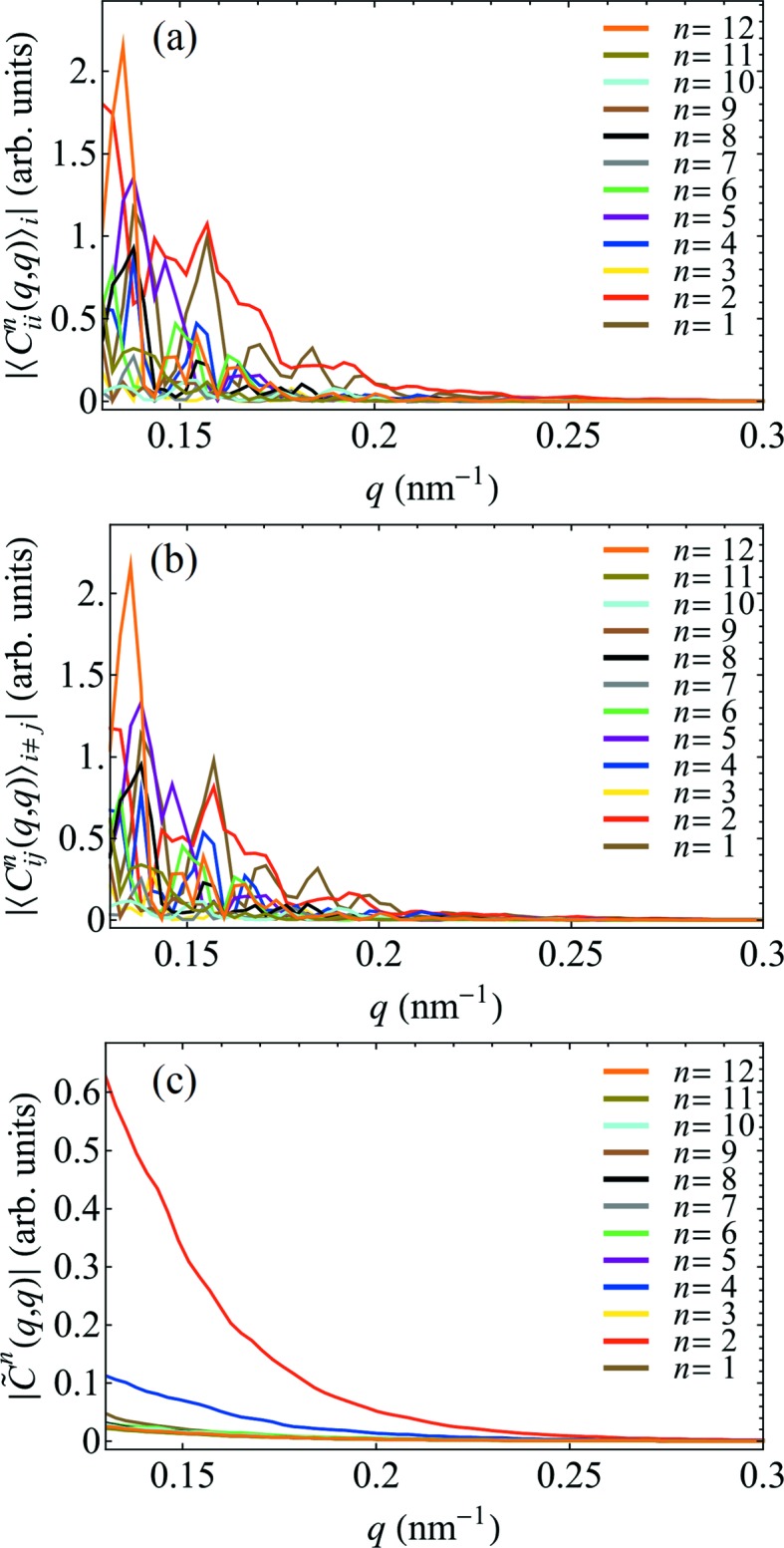
Experimental FXS data calculated for φ_g_ = 0.05% and *T* = 229 K, showing the amplitudes of the FCs of (*a*) the intra-image CCF, 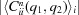, (*b*) the inter-image CCF, 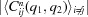, and (*c*) the difference, 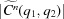 [see equation (17)], determined at *q*_1_ = *q*_2_ = *q* (autocorrelation part of the Fourier spectrum) for *n* = 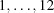.

Fig. 3 ▸ illustrates the importance of utilizing the difference CCF 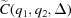 instead of the commonly used CCF 〈*C*_*ii*_(*q*_1_, *q*_2_, Δ)〉_*i*_. As one can see from Fig. 3 ▸, both 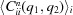 and 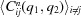 [see equation (17)] have a complex structure with similar magnitudes of even- and odd-order FCs, indicating a strong direct correlation of diffraction patterns which we attribute to an uncompensated and structured background. In contrast, 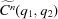 shows a smooth variation of the even-order FCs of interest, as expected for this small-angle scattering experiment.

It is noteworthy that the Fourier spectrum of the autocorrelation 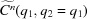 [Fig. 2 ▸(*a*)] differs substantially from the cross-correlation terms^1^ 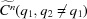 [Figs. 2 ▸(*b*)–2 ▸(*d*)]. This is to be expected due to the contribution of self-correlation of pixels and coherent speckle patterns (Altarelli *et al.*, 2010 ▸; Kurta *et al.*, 2012 ▸, 2013*a* ▸) that are enhanced by autocorrelation (note the bright diagonal streak on all the 2D maps in Fig. 1 ▸). As a result, the contrast of higher-order FCs of the autocorrelation function diminishes rapidly as a function of *q*. In the present case, the FC of the autocorrelation of order *n* = 6 cannot be detected above the background and the FC of the autocorrelation of order *n* = 4 diminishes rapidly, see Fig. 2 ▸(*a*). Yet, the FCs of the CCF of orders *n* = 2, 4 and 6 are clearly dominant across all measured *q* values, see Figs. 2 ▸(*b*)–2 ▸(*d*). Considering a smooth *q* dependence of the amplitudes 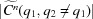 (see Fig. 1 ▸), it is sufficient to analyse the FCs of the CCF determined at one particular *q*_2_ value. Therefore, all further analyses will exclusively involve the cross-correlation terms 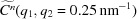.

**Figure 4:**
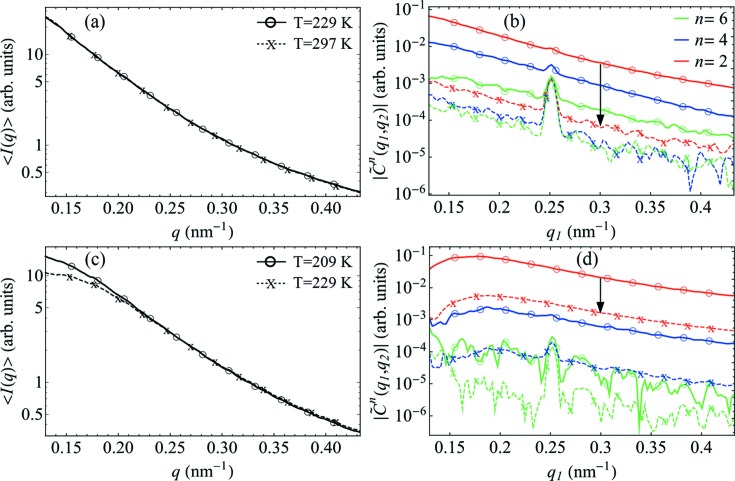
The temperature dependence of the FXS data (log scale). (*a*) and (*c*) The SAXS intensities, and (*b*) and (*d*) the amplitudes of the FCs 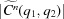 for *n* = 2, 4 and 6 and *q*_2_ = 0.25 nm^−1^, determined at temperatures *T* = 229 and 297 K for the sample with a volume fraction of goethite nanorods φ_g_ = 0.05% [panels (*a*) and (*b*)], and at temperatures *T* = 209 and 229 K for the sample with φ_g_ = 0.5% [panels (*c*)–(*d*)]. The black arrows in panels (*b*) and (*d*) indicate the observed temperature drop of 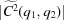.

The temperature dependence of the FXS data is illustrated in Fig. 4 ▸. SAXS intensities [Figs. 4 ▸(*a*) and 4 ▸(*c*)] and the dominant FCs 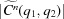 [Figs. 4 ▸(*b*) and 4 ▸(*d*)] are determined for two samples with different volume fractions of goethite particles, φ_g_ = 0.05% [Figs. 4 ▸(*a*) and 4 ▸(*b*)] and φ_g_ = 0.5% [Figs. 4 ▸(*c*) and 4 ▸(*d*)], at different temperatures. For each volume fraction, the data measured at different temperatures were scaled according to equations (1) and (2), assuming the same average number of particles *N* in the beam; see Appendix *C* [App appc] for details of the scaling procedure. The analysis reveals substantial variation in the magnitudes of 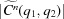 as a function of temperature, while the *q* dependence remains almost unchanged for a particular φ_g_. Indeed, by increasing the solution temperature from *T* = 229 to 297 K (φ_g_ = 0.05%), the dominant FC of order *n* = 2 decreases by almost two orders of magnitude, as indicated by the arrow in Fig. 4 ▸(*b*), while the higher-order FCs vanish completely. Similarly, by increasing the solution temperature in a sample with a higher volume fraction (φ_g_ = 0.5%), the dominant FCs (*n* = 2 and 4) become approximately one order of magnitude smaller, see arrow in Fig. 4 ▸(*d*).

To the best of our knowledge, this is the first reported observation of temperature effects in fluctuation X-ray scattering. It was pointed out by Kam (1977 ▸) that temperature-dependent rotational diffusion of particles during X-ray exposure can smear the intensity fluctuations of diffraction patterns, thus reducing the contrast of the angular CCFs. By reducing the temperature, the viscosity of the solvent increases and rotational diffusion slows down, leading to an increase in contrast. At first glance, the observed temperature behaviour of 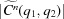 is in agreement with this physical picture. However, as will be shown below, rotational diffusion dynamics is not the decisive factor responsible for the temperature variation in the contrast of 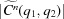 observed in our experiment.

**Figure 5:**
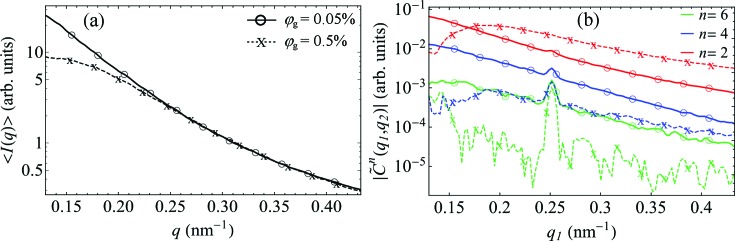
The concentration dependence of the FXS data at *T* = 229 K (log scale). (*a*) The SAXS intensities and (*b*) the amplitudes of the FCs 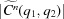 for *n* = 2, 4 and 6 and *q*_2_ = 0.25 nm^−1^, determined for two samples with different volume fractions of goethite nanorods, φ_g_ = 0.5% and 0.05%.

**Figure 6:**
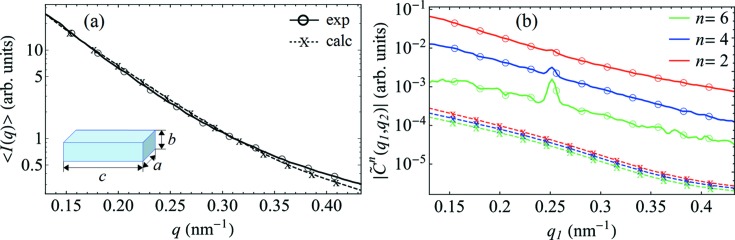
The results of simulations for a polydisperse system of lath-shaped particles [with average dimensions *a* = 23 nm, *b* = 9.2 nm and *c* = 400 nm (see text)] scaled to the experimental FXS data measured at φ_g_ = 0.05% and *T* = 229 K. (*a*) The SAXS intensities and (*b*) the amplitudes 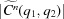 for *n* = 2, 4 and 6 at *q*_2_ = 0.25 nm^−1^. A Gaussian distribution of particle sizes and a uniform distribution of particle orientations were applied in the simulations.

Experimental results for two samples with different volume fractions of goethite particles (φ_g_ = 0.5% and 0.05%) measured at the same temperature (*T* = 229 K) are compared in Fig. 5 ▸. The results were scaled assuming that the number of scattering particles *N* is ten times larger in the sample with the highest volume fraction (φ_g_ = 0.5% compared with φ_g_ = 0.05%). Ideally, in the absence of additional concentration effects the FXS data should overlap after such a rescaling, which is apparently not the case. In the *q* range shown in Fig. 5 ▸, both SAXS intensities and FCs have similar *q* dependencies at different concentrations, but the magnitudes of the FCs are notably different. To understand the origin of this effect we performed simulations of FXS for various model systems (see Appendix *D* [App appd]). We identified a model of the goethite solution that adequately reproduces the SAXS intensity at φ_g_ = 0.05% as well as the *q* dependence of the FCs (Fig. 6 ▸). The model takes particle polydispersity into account and assumes a uniform distribution of orientations, but the simulated FCs have considerably lower values (about three orders of magnitude) than the experimental results [see Fig. 6 ▸(*b*)]. Notably, not only is the entire simulated spectrum shifted down in magnitude, but the relative scaling of simulated FCs at different orders *n* is also different, for instance 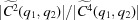 is much larger in the experiment than retrieved in the simulation.

**Figure 7:**
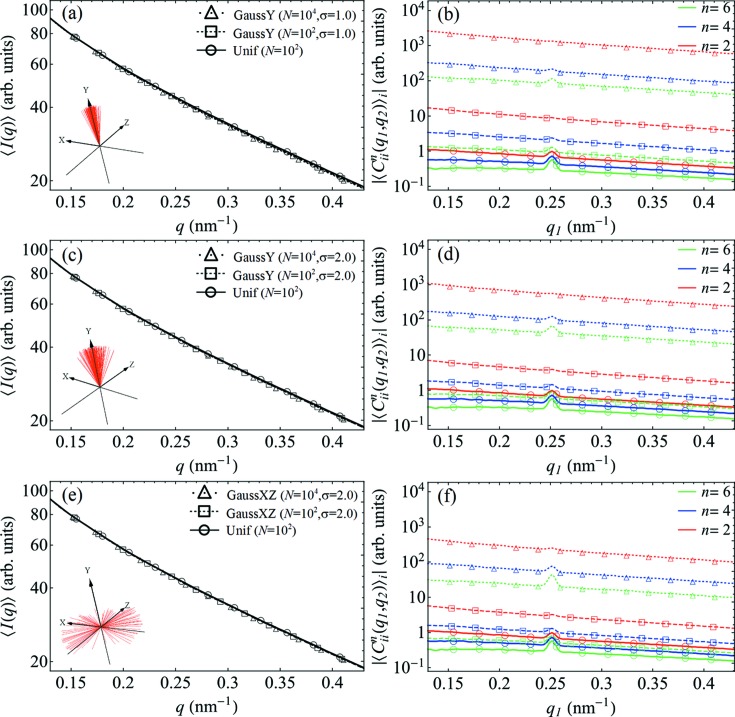
The results of simulations for various types of nonuniform distribution of particle orientations. (*a*), (*c*) and (*e*) The SAXS intensities, and (*b*), (*d*) and (*f*) the amplitudes of the FCs 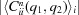 for *n* = 2, 4 and 6 at *q*_2_ = 0.25 nm^−1^. The results for a Gaussian distribution of particle orientations about the *y* axis (GaussY) with σ_*y*_ = 1.0 [panels (*a*) and (*b*)] and σ_*y*_ = 2.0 [panels (*c*) and (*d*)], as well as a Gaussian distribution of orientations about the *xz* plane (GaussXZ) with σ_*xz*_ = 2.0 [panels (*e*) and (*f*)], are plotted together with the results for a uniform distribution (Unif) of orientations. The nonuniform distributions of orientations are shown schematically as insets in panels (*a*), (*c*) and (*e*) (see Appendix *D* [App appd] for details).

**Figure 8:**
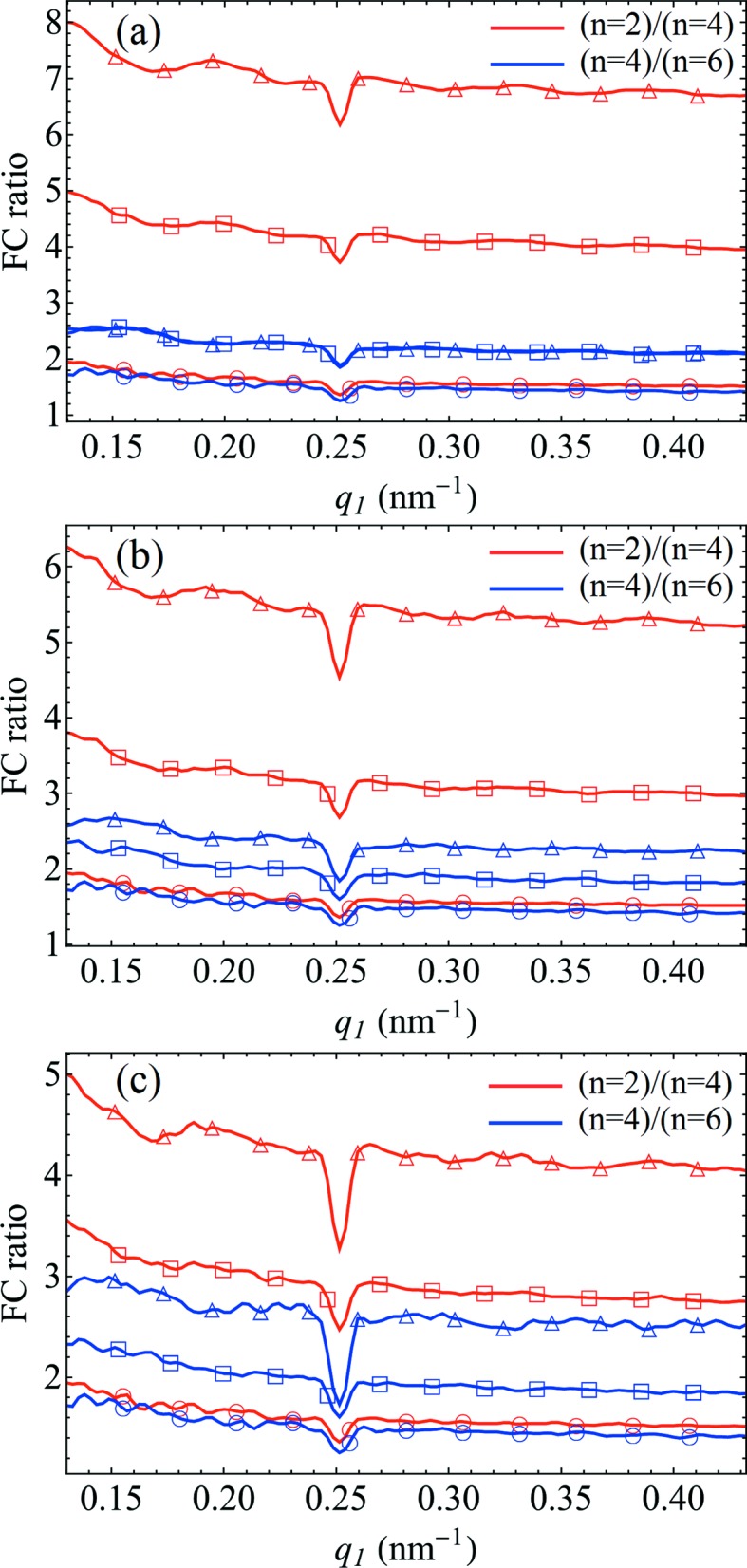
The ratios between the FC amplitudes 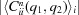 of different orders *n* determined for the model systems shown in Figs. 7 ▸(*a*), 7 ▸(*c*) and 7 ▸(*e*). The FC ratios plotted in panels (*a*), (*b*) and (*c*) were determined between the FCs shown in Figs. 7 ▸(*b*), 7 ▸(*d*) and 7 ▸(*f*), respectively. For instance, the ratio 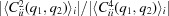 for a model of *N* = 10^4^ particles with a Gaussian distribution of particle orientations about the *y* axis (GaussY) with σ_*y*_ = 1.0 is labelled in panel (*a*) with red triangular markers.

**Figure 9:**
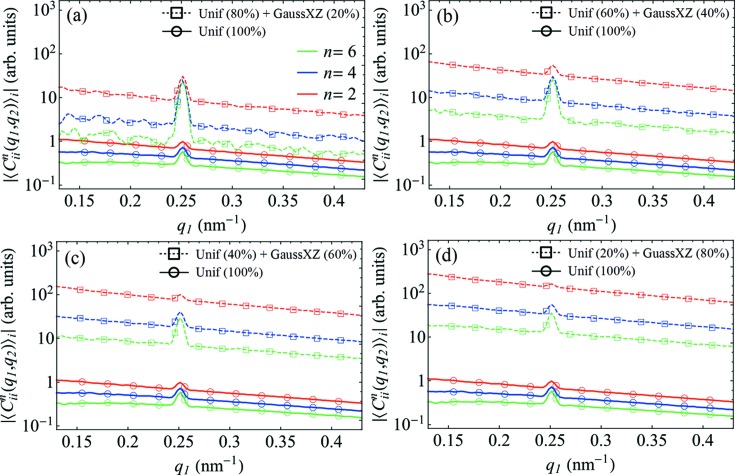
The results of simulations for a partially ordered versus completely disordered system of particles. The amplitudes of the FCs 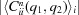 for *n* = 2, 4 and 6 at *q*_2_ = 0.25 nm^−1^ are shown. In the model of a mixed system of particles, a fraction of the particles have a Gaussian distribution of orientations about the *xz* plane (GaussXZ) with σ_*xz*_ = 2.0, while all other particles obey a uniform distribution (Unif), where the corresponding fractions are specified in the figure legend.

Additional simulations have indicated that a nonuniform distribution of particle orientations may be responsible for the observed effects (see Figs. 7 ▸ and 9 ▸). Our results show that the FCs of the CCF have substantially higher values in the case of a Gaussian distribution of orientations around a mean direction, and for arbitrarily large numbers of particles they can be several orders of magnitude larger than for a perfectly uniform distribution. Importantly, in the case of a nonuniform distribution of particle orientations, the relative scaling of FCs of different orders *n* is also affected, closely resembling what is observed in the experiment (see Fig. 8 ▸). Similar effects can be observed for models where only a fraction of the particles obey a nonuniform distribution of orientations while the others exhibit truly random orientations (see Fig. 9 ▸).

While the details depend on the particular parameters of the nonuniform orientational distribution, the simulations generally indicate a nonlinear dependence of the FCs on the number of particles *N* in the system, meaning that equation (2) does not hold in this case. Such a nonlinear scaling of FCs was deduced earlier for a 2D disordered system of particles with a Gaussian distribution of orientations about a certain direction (Kurta *et al.*, 2012 ▸). It has been shown that the *N*-dependent scaling factor for an FC of *n*th order is equal to 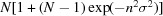, where σ is the standard deviation of the Gaussian distribution. In the limit of σ → ∞ this expression tends towards *N*, which is the exact result for a uniform distribution^2^. This means that for a nonuniform distribution, FCs of different orders have different scaling parameters which depend in a nonlinear fashion on *N*. Clearly, in the 3D case relevant for our experiment, a specific non­uniform distribution of orientations could further alter the *q* dependence and relative scaling of individual FCs. While it is not feasible to obtain a general analytical result for an arbitrary non-uniform distribution of particle orientations in 3D [similar to equation (2)], simulations can still provide valuable information for qualitative analyses.

**Figure 10:**
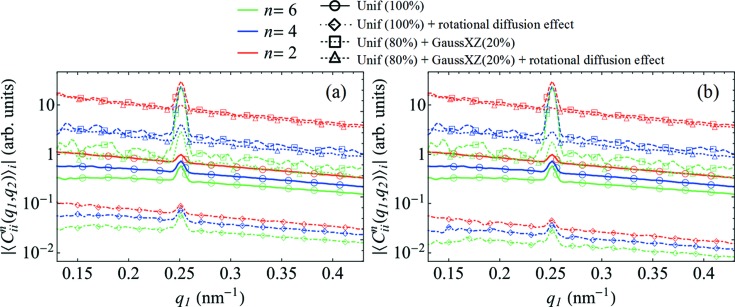
The results of simulations of rotational diffusion effects in systems with and without orientational order. The amplitudes of the FCs 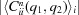 for *n* = 2, 4 and 6 at *q*_2_ = 0.25 nm^−1^ are shown. In the model of a mixed system of particles, a fraction of the particles have a Gaussian distribution of orientations about the *xz* plane (GaussXZ) with σ_*xz*_ = 2.0, while all other particles obey a uniform distribution (Unif), where the corresponding fractions are specified in the figure legends. The results of simulations with rotational diffusion are compared with those without rotational diffusion [shown in Fig. 9 ▸(*a*)]. The difference between panels (*a*) and (*b*) is in the distinct number of X-ray snapshots, (*a*) ten versus (*b*) 20, averaged to form individual averaged diffraction patterns, when simulating rotational diffusion effects (see Appendix *E* [App appe]).

Taking into account the devised model of nanoparticle solution studied in our experiment, which involves orientational nonuniformity of particles, we can finally discuss possible origins of the observed temperature variation of 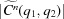, particularly the role of rotational diffusion of particles. Our simulations show (see Appendix *E* [App appe] and Fig. 10 ▸) that, in the case of orientational order of particles, the rotational diffusion dynamics has a rather minor effect on the contrast of 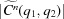. Therefore, temperature-dependent orientational correlations of particles are predominantly responsible for the observed FXS contrast variation. The data available from our experiment make it difficult to distinguish whether direct particle–particle interactions or particle–wall interactions are responsible for these correlations. Considering the very low sample volume fraction, however, we are inclined to interpret our observations as a result of inadequate equilibration (due to the high solvent viscosity at low temperature) after sample loading, or an alignment effect induced by the capillary walls. A more systematic study is required to identify uniquely the physical origin of the observed temperature-dependent orientational correlations.

## 5. Conclusions and outlook   

Fluctuation scattering based on higher-order statistics of the scattered intensities, combined with novel capabilities of X-ray instrumentation and advanced approaches to data analysis, opens up exciting new opportunities for materials research with X-rays. Our results indicate that comprehensive information about the structure and dynamics of disordered systems can be extracted by means of angular cross-correlation functions. We have shown that FXS from solutions of nanoparticles can provide unique structural information, which is challenging or impossible to obtain by conventional SAXS approaches.

The ‘classical’ aim of FXS formulated by Kam (1977 ▸) is to use the angular CCFs as additional constraints in the particle structure determination problem. Such studies rely on a uniform distribution of particle orientations in solution, which is a prerequisite of a successful 3D reconstruction. However, the experimentally measured CCFs can be affected by two major factors, namely rotational diffusion dynamics and inter-particle interactions, leading to quite distinct outcomes for the FXS data.

According to earlier theoretical predictions for isotropic solutions (Kam, 1977 ▸; Kam *et al.*, 1981 ▸), temperature-dependent rotational diffusion of particles during X-ray exposure can smear the intensity fluctuations of the diffraction pattern, thus reducing the contrast of the angular CCFs. Therefore, particle dynamics prevents structural information being accessed by FXS because the higher-order scattering terms vanish. To solve the problem it was suggested to cool the solution down in order to slow down the dynamics due to the resulting increase in solvent viscosity (Kam, 1977 ▸; Kam *et al.*, 1981 ▸). The effect of rotational diffusion is naturally diminished at novel X-ray sources like X-ray free-electron lasers (XFELs) and diffraction-limited storage rings, where extremely high numbers of photons can be delivered to the sample in ultrashort pulses, much shorter than the characteristic rotational diffusion times of materials. This makes such X-ray sources well suited to structural characterization by FXS. On the other hand, angular cross-correlation functions provide a new tool for studying rotational diffusion dynamics that is notoriously difficult to access experimentally.

Interparticle interactions can introduce distortions in the angular CCFs, which may prevent them being used for particle 3D structure recovery. In conventional SAXS structural studies, the role of interparticle interactions can be effectively diminished by reducing the sample concentration, resulting in SAXS intensity curves that can be used directly for structure refinement. The FXS data appear to be much more sensitive to nonuniformities in the orientational distribution of particles, which manifest themselves in the measured CCFs even at subtle deviations from the isotropic case, in contrast to SAXS. Other sources of orientational nonuniformities may be induced by external forces (optical excitations, magnetic fields *etc*), the sample container (*e.g.* particle–wall interaction) or solute flow alignment. Therefore, the possibility of unwanted orientational ordering should be carefully considered in all experiments aimed at single-particle structure recovery from solution scattering, because the resulting nonlinear and non-trivial scaling of higher-order scattering terms will distort the structural information obtained. On the other hand, FXS may be considered as a tool to study weak orientational order and correlations in solutions, where other methods cannot provide the desired sensitivity.

In our experimental study, by varying the temperature of the goethite nanorod solution, we observed substantial changes in the FXS contrast. We also revealed significant deviations of the correlated scattering from that expected for an isotropically oriented sample. Our simulations show that nonuniformities in the orientational distribution of goethite particles may be responsible for the observed features in FXS. These results demonstrate that FXS can also be used as a sensitive probe of orientational alignment in apparently disordered systems, which is an essential capability for nanoscale studies of inhomogeneities, cooperativity and early stages of nucleation in solutions. The sample model involving orientational correlations also suggests that rotational diffusion dynamics plays a minor role in the temperature-dependent variation of the FXS contrast. In the present case, the goethite solutions are believed to be isotropic in bulk at low concentrations (≤4%), so the weak anisotropy observed is probably an artifact from the sample loading or an alignment effect induced by the capillary walls. In either case, FXS demonstrates extreme sensitivity to weak nematic ordering of anisotropic particles.

## Footnotes

1 Peaks in  visible in Figs. 2 ▸(*b*)–2 ▸(*d*) appear when the corresponding section shown in Fig. 1 ▸(*d*) crosses the diagonal *q*_1_ = *q*_2_. These peaks appear due to autocorrelation of intensity at the same *q* and should not be considered as cross-correlation terms defined strictly at *q*_1_ ≠ *q*_2_; the magnitudes of  at the peak positions are the same as in Fig. 2 ▸(*a*) at the corresponding *q* = *q*_2_ value.

2 The scaling factor given by Kurta *et al.* (2012 ▸) is specified for a normalized two-point CCF and should be multiplied by *N* ^2^ in order to get the expression shown here.

## Appendix A Two-point cross-correlation function for a dilute polydisperse mixture of particles

We consider X-ray scattering from a single particle and define single-particle scattered intensity and the cross-correlation function (CCF). The scattered intensity distribution from a single particle of the *p*th kind in an arbitrary *k*th orientation can be expressed as

where **q** is the scattering vector, **r** is the real-space vector and ρ_*p*, *k*_(**r**) is the 3D electron-density distribution of the particle of the *p*th kind in the *k*th orientation. *I*_*p*, *k*_(**q**) can be expanded into an angular Fourier series as

where the angular Fourier transform is defined in the polar coordinate system **q** = (*q*, φ) and  are the Fourier components (FCs) of *I*_*p*, *k*_(*q*, φ).

The two-point angular cross-correlation function^3^ (CCF) is defined at two momentum transfer values *q*_1_ and *q*_2_ as (Kam, 1977 ▸; Kurta *et al.*, 2013*a* ▸, 2016 ▸)

where Δ is the angular separation and  defines averaging over the angular coordinate φ, with the subscript *p* indicating that the CCF is for a particle of the *p*th kind in the *k*th orientation. It is customary to operate with the FCs  of the CCF (5) with angular Fourier series of *C*_*p*, *k*_(*q*_1_, *q*_2_, Δ) written as (Altarelli *et al.*, 2010 ▸)

It has been shown that the following relation holds between the FCs of the intensity and of the CCF (Altarelli *et al.*, 2010 ▸)

As one can see, the FCs of the CCF are determined by the FCs of the intensities  and .

The CCF and its FCs can be averaged over a set of *M* diffraction patterns to obtain orientationally averaged results,

where  denotes statistical averaging.

Now we consider X-ray scattering from a dilute mixture of particles consisting of *N*_*s*_ different species. In this case the scattered intensity distribution (neglecting particle interference terms and hence valid for dilute suspensions) recorded on the *i*th detector image can be expressed as

where the first summation is performed over *N*_*s*_ different particle types, the second summation is done over *N*_*p*_ particles of each particle type *p* (the value of *N*_*p*_ depends on *p*), and intensities  are defined by equation (3) for each particle type *p* and orientation *k*_*p*_. Here we have also introduced the experimental scaling factor *A*_*i*_, which can vary from image to image. For the sake of simplicity, we have assumed in equation (9) that *A*_*i*_ is a **q**-independent quantity mostly defined by the incident X-ray flux, the beam footprint and the sample exposure time (or pulse duration), and that each measured scattered intensity distribution is corrected for **q**-dependent experimental factors (*e.g.* polarization and detector geometry). Similar to equation (5), we can define the CCF *C*_*i*_(*q*_1_, *q*_2_, Δ) in terms of *I*_*i*_(*q*, φ) as

Assuming a uniform distribution of particle orientations, statistically averaged CCFs 〈*C*_*i*_(*q*_1_, *q*_2_, Δ)〉_*i*_ and their FCs  can be expressed as

where the single-particle quantities 〈*C*_*p*, *i*_(*q*_1_, *q*_2_, Δ)〉_*i*_ and  are defined in equations (8*a*) and (8*b*), respectively. In the above expressions we have assumed that fluctuations in the experimental factor *A*_*i*_ and in the number of particles *N*_*p*, *i*_ attributed to each scattering image are statistically independent quantities.

As one can see from equations (11*a*) and (11*b*), in the case of a mixture of particles the statistically averaged CCFs and their FCs can be represented as a sum of single-particle quantities weighted by the average number of particles 〈*N*_*p*, *i*_〉_*i*_ of each kind *p* in the mixture. This result is similar to that obtained for two-dimensional heterogeneous disordered ensembles of particles (Chen *et al.*, 2013 ▸). An important difference between the 2D case and the 3D case considered here is that for the latter the ensemble-averaged quantities  cannot be further factorized in terms of intensity FCs [similar to equation (7)], while this is still possible in the 2D case.

For a polydisperse mixture of particles, the CCF 〈*C*_*i*_(*q*_1_, *q*_2_, Δ)〉_*i*_ and its FCs  can be defined as follows:

where the integration is performed over all particle sizes *s* and for which a normalized particle size distribution function is defined:  = 1. *N* =  is the total number of particles in the illuminated sample volume and the single-particle quantities 〈*C*_*s*, *i*_(*q*_1_, *q*_2_, Δ)〉_*i*_ and  are defined for each particle size according to equations (8*a*) and (8*b*).

For completeness, we also specify a small- (or wide-)angle scattering (SAXS or WAXS) intensity for a polydisperse system of particles as

where 〈*I*_*s*, *i*_(*q*)〉_*i*_ is the SAXS intensity determined for a particle of size *s*,

and *I*_*s*, *i*_(*q*, φ) is defined by equation (3), where the subscripts *p*, *k* should be replaced by *s*, *i*, respectively. Equations (13) and (12*b*) contain key expressions used for FXS data interpretation in this work [see equations (1) and (2) in the main text].

In the present manuscript we have employed a Fourier basis for expressing the scattered intensity and cross-correlation functions. Using a Fourier basis is typically preferred when considering scattering from 2D systems, or in the case of small-angle scattering with negligible curvature of the Ewald sphere. At the same time, a Legendre basis is more natural in the case of wide-angle scattering from 3D objects, particularly enabling more straightforward treatment of effects associated with curvature of the Ewald sphere.

## Appendix B Intra- and inter-image cross-correlation functions

For practical applications, it is helpful to determine the so-called difference CCF (Kurta *et al.*, 2014 ▸, 2017 ▸)

and its Fourier components defined in the series

where *C*_*ii*_(*q*_1_, *q*_2_, Δ) is the CCF determined on the same *i*th image [intra-image CCF, commonly used in the literature (Kam, 1977 ▸; Kurta *et al.*, 2013*a* ▸, 2016 ▸)] and *C*_*ij*_(*q*_1_, *q*_2_, Δ) defines the angular correlation between two different images *i* ≠ *j* (inter-image CCF). Due to the linear properties of the Fourier transformation, the average difference Fourier spectrum can be also determined as (Kurta *et al.*, 2014 ▸, 2017 ▸)

where the second term on the right-hand side is defined strictly for *i* ≠ *j*.

In the case of nonuniformity of the measured data (*e.g.* structured background, nonuniform response of detector *etc.*), calculating the difference FCs  helps significantly in reducing artifacts that otherwise contaminate .

## Appendix C Comparison of the FXS data sets

Here we describe the procedure for comparing FXS data sets, which can be applied to test modelling against experimental data or to compare different experimental results. In general, precise information about the scaling factors *A* in equations (1) and (2) is required to compare different data sets. In the case of a uniform distribution of orientations, both the SAXS intensity and the FCs of the CCF depend linearly on the number of particles *N*, while the dependence of the experimental scaling factor is linear or quadratic, respectively. Note that generally 〈*A* ^2^〉 ≥ 〈*A*〉^2^, but the approximation 〈*A* ^2^〉 ≃ 〈*A*〉^2^ can be applied if for each diffraction pattern *A* = 〈*A*〉 + δ*A*, and δ*A* is an arbitrarily small fluctuation about the average value 〈*A*〉 (δ*A*  〈*A*〉, 〈δ*A*〉 = 0). This approximation significantly simplifies direct comparison of the FXS data and is valid, for example, if the incident intensity fluctuates slightly about some average value. In practice, experimental scattering patterns can be normalized by the measured incident intensities before further analysis. In the absence of accurately measured incident intensities, the effect of incident intensity fluctuations can be reduced by normalizing the intensity of each diffraction pattern by its average intensity per pixel *I*_av_ (*I*_av_ = , where *I*_*j*_ is the intensity value of the *j*th pixel and summation is done over *p* pixels). Simulations show that the applicability of such a normalization approach is justified if the statistical properties of the sample, *e.g.* the probability distribution of particle orientations, are preserved during measurements. This normalization procedure was applied here.

Let us consider the following example of validating a model against experimental FXS data. In simulations we assume a particular form of the particle size distribution function *D*(*s*), with a defined particle structure for each size *s*. Suppose that we know the illuminated sample volume *V*_sample_ and the particle volume fraction φ_g_ (%). Then the number of scattering particles *N*_exp_ is given by

where *V*(*s*) is the volume of a particle of size *s* in the considered model. Scaling of the simulated SAXS intensity 〈*I*(*q*)〉_sim_ to the experimental value 〈*I*(*q*)〉_exp_ is then performed by first multiplying 〈*I*(*q*)〉_sim_ by *N*_sc_ = *N*_exp_/*N*_sim_, where *N*_sim_ is the number of particles used in the simulation, thus yielding an updated (partially scaled) value of 〈*I*(*q*)〉_sim_. The scaling ratio *A*_sc_ = 〈*A*〉_exp_/〈*A*〉_sim_ = 〈*I*(*q*)〉_exp_/〈*I*(*q*)〉_sim_ is determined using this updated value. The magnitude of *A*_sc_ should not depend on the choice of *q* so it is reasonable to calculate the ratio 〈*I*(*q*)〉_exp_/〈*I*(*q*)〉_sim_ at a *q* value where the experimental data have the best signal-to-noise ratio. We then multiply the simulated SAXS intensity by *A*_sc_ and the simulated FCs by  (using the approximation 〈*A* ^2^〉 ≃ 〈*A*〉^2^ discussed above). After this step, the rescaled simulated SAXS intensity and FCs allow a direct comparison with the experimental data to test the accuracy of the model.

In equations (1) and (2) we assumed that the particle orientations are uniformly distributed (isotropic case). If this is not the case, equations (1) and (2) do not hold and the above scaling procedure is also wrong, in particular because the *N*-dependent factors have more complicated forms. It has been shown that, for a partially disordered 2D ensemble of particles specified by a Gaussian distribution of orientations with standard deviation σ, the scaling factor in equation (2) becomes equal to , in contrast to *N* in the case of a uniform distribution (Kurta *et al.*, 2012 ▸). In 3D simulations of nonuniform orientation distributions of goethite nanorods, we also observed nonlinear *N* dependencies of CCFs and FCs of different orders *n*. Therefore, if a discrepancy is present after rescaling the model this may indicate nonuniformities in the orientational distribution of particles, which is an interesting topic in itself (see simulation results in Appendix *D* [App appd]).

## Appendix D Simulations of FXS

We performed FXS simulations by employing bead models of various structures within a uniform density approximation. The X-ray scattering simulations were performed with parameters identical to those of the experiment, *i.e.* a photon energy of 10 keV and a 2D detector consisting of 256 × 256 square pixels (55 μm pixel size) placed 515 mm downstream from the sample. In all simulations we considered a fully coherent beam and did not include shot noise. In the absence of any structured background and detector artifacts, it is sufficient to compute the commonly used CCF 〈*C*_*ii*_(*q*_1_, *q*_2_, Δ)〉_*i*_ (see Appendix *B* [App appb]) and analyse its FCs  for every scattering image *i* in order to obtain all necessary structural information.

In the simulations a lath-shaped structure of goethite nanoparticles was assumed, as illustrated in the inset of Fig. 6 ▸(*a*). Solvent scattering was neglected due to its negligible scattering contribution compared with the strong signal from goethite. To model the particle polydispersity, we assumed a truncated Gaussian distribution of sizes, reported earlier for goethite in solution (Lemaire *et al.*, 2004 ▸). The particle size distribution function *D*(*s*) was effectively defined as a 2D Gaussian function with mean values *M*_*a*_, *M*_*c*_ and corresponding standard deviations σ_*a*_, σ_*c*_ of the particle dimensions *a* and *c*, respectively. The third particle dimension was defined as *b* = *a*/*p* for each value of *a*, where *p* is an anisotropy parameter. Due to the low experimental resolution and particle polydispersity, our model fits showed limited sensitivity to the largest particle dimension. However, the best fitted model to the experimental SAXS intensity at φ_g_ = 0.05% and *T* = 229 K [see Fig. 6 ▸(*a*)] has *p* = 2.5, *M*_*a*_ = 23 nm, *M*_*c*_ = 400 nm, σ_*a*_ = 20.5 nm and σ_*c*_ = 95 nm, which are close to earlier reported values (Poulos *et al.*, 2010 ▸).

With these parameters, the model contains about *N*_exp_ = 0.7 × 10^6^ particles in the illuminated volume at φ_g_ = 0.05%, estimated according to equation (18). This value of *N*_exp_ was used to rescale the simulated FXS data to the experimental results shown in Fig. 6 ▸ (see Appendix *C* [App appc] for the scaling procedure). The SAXS intensities and the *q* dependence of the FCs  are reproduced reasonably well, although the simulated FCs have considerably lower magnitudes than the experimental results. An inaccurate estimation of the number of particles can only be partially responsible for this significant discrepancy and other causes must be sought.

Our simulations show that nonuniformities in the orientational distribution of particles may in fact be responsible for the observed effects. In the case of a uniform distribution of particle orientations, equation (2) can be expressed as an integral over the values of , where  is a single-particle quantity. This is not the case for an arbitrary nonuniform distribution of particle orientations in 3D, and one has to perform full-size simulations with a large number of particles to obtain a precise estimation of the effect. For a qualitative demonstration, we deploy a simplified model and perform simulations of nonuniform distributions of particle orientations for a system of monodisperse lath-shaped particles with dimensions *a* = 7.7 nm, *b* = 3 nm and *c* = 100 nm.

The results of simulations for various types of truncated^4^ Gaussian distribution of particle orientations are compared with those for a uniform distribution of orientations in Fig. 7 ▸. The ‘particle orientation’ was defined as the orientation of the long axis (*c*). We consider Gaussian distributions of particle orientation along the *y* axis, which is perpendicular to the incident X-ray beam [Figs. 7 ▸(*a*)–7 ▸(*d*)] with σ_*y*_ = 1.0 [Figs. 7 ▸(*a*) and 7 ▸(*b*)] and σ_*y*_ = 2.0 [Figs. 7 ▸(*c*) and 7 ▸(*d*)]. A Gaussian distribution of particle tilts about the *xz* plane oriented perpendicular to the detector plane with σ_*xz*_ = 2.0 [Figs. 7 ▸(*e*) and 7 ▸(*f*)] was also considered. In the latter case, particle orientations were generated as follows. First aligned with its long axis parallel to the *z* axis (parallel to the incident-beam direction), the particle was rotated about the *z* axis by a random angle in the range [0, 2π], then rotated about the *x* axis by an angle defined according to the specified Gaussian distribution in the range [−π/2, π/2], and finally rotated about the *y* axis by a random angle in the range [0, 2π]. Calculations were performed for a different number of particles in the sample (specified in the legends in Fig. 7 ▸) and scaled to the results for a uniform distribution of particles. We applied the scaling procedure described in Appendix *C* [App appc] to show how nonuniformities in the orientational distribution of particles can be revealed by comparing the results with those for a uniform distribution of orientations.

As one can see from Fig. 7 ▸, while in all considered cases the rescaled SAXS intensities match precisely, the FCs  always have higher values for models with a nonuniform distribution of particle orientations. The discrepancy between the results for uniform and Gaussian distributions of particles increases substantially as the number of particles in the system grows and can reach several orders of magnitude. Importantly, the relative scaling of FCs of different orders *n* also increases compared with the model with a uniform distribution of particles (see Fig. 8 ▸). Both features are consistent with our experimental observations, suggesting that an anisotropic orientational distribution of particles may be responsible for the observed effects.

The simulation results in Fig. 9 ▸ show that the FCs  can help to reveal even arbitrarily weak orientational order. Here, we simulated X-ray scattering from a mixture of *N* = 10^4^ particles, where a fraction of them are characterized by a Gaussian distribution of particle orientations about the *xz* plane with σ_*xz*_ = 2.0 but the rest are uniformly oriented. Again, the results are scaled to those for a system of *N* = 100 uniformly oriented particles. Obviously, even for a particle model with only 20% obeying a Gaussian distribution of orientations around a specific direction [Fig. 9 ▸(*a*)] the scaled FC of order *n* = 2 appears to be ten times higher than the uniform case.

Note that in all simulated Fourier spectra  the autocorrelation peak is present at *q* = 0.25 nm^−1^ that is the result of coherent interference of interparticle scattering. However, such a peak is absent in the simulated FCs shown in Fig. 6 ▸(*b*), which were determined according to equation (2). In this particular case the X-ray scattering simulations were performed on single particles, in order to calculate CCFs  for each particle size *s*. Since interparticle interference effects were absent from these simulations, this resulted in the absence of the autocorrelation peak in the Fourier components .

## Appendix E Simulations of rotational diffusion effects in FXS

Here we show how the rotational diffusion dynamics of particles in solution can effect the results of correlation analysis. As a starting point we use two simple models, the first with a uniform distribution of particle orientations and the second representing a partially ordered sample, where a fraction of the particles (20%) are characterized by a Gaussian distribution of orientations about the *xz* plane with σ_*xz*_ = 2.0 and the rest are uniformly oriented (80%). The results of correlation analysis for these two models (without rotational diffusion effects included) are shown in Fig. 9 ▸(*a*).

To give a qualitative demonstration of the effects associated with rotational diffusion of particles during X-ray exposure, we applied averaging of simulated diffraction patterns determined for a sequence of instantaneous sample configurations (X-ray snapshots of the sample). In this manner, new sets of averaged diffraction patterns were formed where intensity fluctuations were smeared out to certain degree (depending on the number of patterns in the average), thus simulating the effect of contrast smearing due to rotational diffusion. These new sets of averaged patterns determined for each sample model were used to perform angular correlation analysis.

The results of simulations for models with and without rotational diffusion effects included are presented in Fig. 10 ▸. The difference between Figs. 10 ▸(*a*) and 10 ▸(*b*) is in the distinct number of snapshots averaged to form individual average diffraction patterns in the new data sets, *M*_1_ = 10 in Fig. 10 ▸(*a*) and *M*_2_ = 20 in Fig. 10 ▸(*b*). In the calculations of CCFs, 1000 average patterns were used to produce the results in Fig. 10 ▸(*a*) and 500 patterns in Fig. 10 ▸(*b*). In a real experiment this would correspond to a faster rotational diffusion dynamics for the results shown in Fig. 10 ▸(*b*) than for those in Fig. 10 ▸(*a*). As one can see from the obtained results, after adding rotational diffusion effects to the simulations, the contrast between the FCs decreases fast in the case of a solution with a uniform distribution of particles, in agreement with theoretical predictions (Kam, 1977 ▸). At the same time, the values of the FCs  for a system with partial orientational order remain almost unaffected upon the inclusion of rotational diffusion effects. This is a logical result, since in spite of the smearing of individual intensity fluctuations associated with instantaneous particle positions and orientations, the orientational distribution of particles remains (on average) the same and forms the major contrast in the measured diffraction patterns. This means that rotational diffusion dynamics do not play a significant role if particle orientations maintain the same nonuniform probability distribution of orientation during measurements. We would like to note that our simplified model can only qualitatively explain the dependency of FXS contrast on rotational diffusion. More realistic simulations should be based on a real thermodynamic model of the system.

## References

[bb1] Ackerson, B. J., Taylor, T. W. & Clark, N. A. (1985). *Phys. Rev. A*, **31**, 3183–3193. 10.1103/physreva.31.3183 9895872

[bb2] Als-Nielsen, J. & McMorrow, D. (2011). *Elements of Modern X-ray Physics*, 2nd ed. Hoboken: John Wiley & Sons Inc.

[bb3] Altarelli, M., Kurta, R. P. & Vartanyants, I. A. (2010). *Phys. Rev. B*, **82**, 104207.

[bb4] Chen, G., Modestino, M. A., Poon, B. K., Schirotzek, A., Marchesini, S., Segalman, R. A., Hexemer, A. & Zwart, P. H. (2012). *J. Synchrotron Rad.* **19**, 695–700. 10.1107/S0909049512023801 22898947

[bb5] Chen, G., Zwart, P. H. & Li, D. (2013). *Phys. Rev. Lett.* **110**, 195501. 10.1103/PhysRevLett.110.195501 23705716

[bb6] Clark, N. A., Ackerson, B. J. & Hurd, A. J. (1983). *Phys. Rev. Lett.* **50**, 1459–1462.

[bb7] Donatelli, J. J., Zwart, P. H. & Sethian, J. A. (2015). *Proc. Nat. Acad. Sci. USA*, **112**, 10286–10291. 10.1073/pnas.1513738112 PMC4547282 26240348

[bb8] Kam, Z. (1977). *Macromolecules*, **10**, 927–934.

[bb9] Kam, Z., Koch, M. H. J. & Bordas, J. (1981). *Proc. Nat. Acad. Sci. USA*, **78**, 3559–3562. 10.1073/pnas.78.6.3559 PMC319609 6943555

[bb10] Kirian, R. A., Schmidt, K. E., Wang, X., Doak, R. B. & Spence, J. C. H. (2011). *Phys. Rev. E*, **84**, 011921. 10.1103/PhysRevE.84.011921 21867227

[bb11] Kurta, R. P. (2016). *J. Phys. B At. Mol. Opt. Phys.* **49**, 165001.

[bb12] Kurta, R. P., Altarelli, M. & Vartanyants, I. A. (2013*a*). *Adv. Cond. Matt. Phys.* **2013**, 959835.

[bb13] Kurta, R. P., Altarelli, M. & Vartanyants, I. A. (2016). *Adv. Chem. Phys.* **161**, 1–39.

[bb14] Kurta, R. P., Altarelli, M., Weckert, E. & Vartanyants, I. A. (2012). *Phys. Rev. B*, **85**, 184204.

[bb19] Kurta, R. P., Donatelli, J. J., Yoon, C. H., Berntsen, P., Bielecki, J., Daurer, B. J., DeMirci, H., Fromme, P., Hantke, M. F., Maia, F. R. N. C., Munke, A., Nettelblad, C., Pande, K., Reddy, H. K. N., Sellberg, J. A., Sierra, R. G., Svenda, M., van der Schot, G., Vartanyants, I. A., Williams, G. J., Xavier, P. L., Aquila, A., Zwart, P. H. & Mancuso, A. P. (2017). *Phys. Rev. Lett.* **119**, 158102. 10.1103/PhysRevLett.119.158102 PMC5757528 29077445

[bb15] Kurta, R. P., Dronyak, R., Altarelli, M., Weckert, E. & Vartanyants, I. A. (2013*b*). *New J. Phys.* **15**, 013059.

[bb16] Kurta, R. P., Grodd, L., Mikayelyan, E., Gorobtsov, O. Y., Fratoddi, I., Venditti, I., Sprung, M., Grigorian, S. & Vartanyants, I. A. (2014). *J. Phys. Conf. Ser.* **499**, 012021. 10.1039/c5cp00426h 25700131

[bb17] Kurta, R. P., Grodd, L., Mikayelyan, E., Gorobtsov, O. Y., Zaluzhnyy, I. A., Fratoddi, I., Venditti, I., Russo, M. V., Sprung, M., Vartanyants, I. A. & Grigorian, S. (2015). *Phys. Chem. Chem. Phys.* **17**, 7404–7410. 10.1039/c5cp00426h 25700131

[bb18] Kurta, R. P., Ostrovskii, B. I., Singer, A., Gorobtsov, O. Y., Shabalin, A., Dzhigaev, D., Yefanov, O. M., Zozulya, A. V., Sprung, M. & Vartanyants, I. A. (2013*c*). *Phys. Rev. E*, **88**, 044501. 10.1103/PhysRevE.88.044501 24229307

[bb20] Lehmkühler, F., Grübel, G. & Gutt, C. (2014). *J. Appl. Cryst.* **47**, 1315–1323.

[bb21] Lemaire, B. J., Davidson, P., Ferré, J., Jamet, J. P., Petermann, D., Panine, P., Dozov, I. & Jolivet, J. P. (2004). *Eur. Phys. J. E*, **13**, 291–308. 10.1140/epje/i2003-10078-6 15103523

[bb22] Lhermitte, J. R., Tian, C., Stein, A., Rahman, A., Zhang, Y., Wiegart, L., Fluerasu, A., Gang, O. & Yager, K. G. (2017). *J. Appl. Cryst.* **50**, 805–819.

[bb23] Liu, A. C. Y., Lumpkin, G. R., Petersen, T. C., Etheridge, J. & Bourgeois, L. (2015). *Acta Cryst.* A**71**, 473–482. 10.1107/S2053273315011845 26317191

[bb24] Liu, A. C. Y., Neish, M. J., Stokol, G., Buckley, G. A., Smillie, L. A., de Jonge, M. D., Ott, R. T., Kramer, M. J. & Bourgeois, L. (2013*a*). *Phys. Rev. Lett.* **110**, 205505. 10.1103/PhysRevLett.110.205505 25167428

[bb25] Liu, A. C. Y., Tabor, R. F., Bourgeois, L., de Jonge, M. D., Mudie, S. T. & Petersen, T. C. (2016). *J. Stat. Mech.* **2016**, 054046.

[bb26] Liu, H., Poon, B. K., Janssen, A. J. E. M. & Zwart, P. H. (2012). *Acta Cryst.* A**68**, 561–567. 10.1107/S0108767312029637 22893239

[bb27] Liu, H., Poon, B. K., Saldin, D. K., Spence, J. C. H. & Zwart, P. H. (2013*b*). *Acta Cryst.* A**69**, 365–373. 10.1107/S0108767313006016 23778093

[bb28] Malmerberg, E., Kerfeld, C. A. & Zwart, P. H. (2015). *IUCrJ*, **2**, 309–316. 10.1107/S2052252515002535 PMC4420540 25995839

[bb29] Mancini, G. F., Latychevskaia, T., Pennacchio, F., Reguera, J., Stellacci, F. & Carbone, F. (2016). *Nano Lett.* **16**, 2705–2713. 10.1021/acs.nanolett.6b00355 26918756

[bb30] Martin, A. V. (2017). *IUCrJ*, **4**, 24–36. 10.1107/S2052252516016730 PMC5331463 28250939

[bb31] Mendez, D., Lane, T. J., Sung, J., Sellberg, J., Levard, C., Watkins, H., Cohen, A. E., Soltis, M., Sutton, S., Spudich, J., Pande, V., Ratner, D. & Doniach, S. (2014). *Philos. Trans. R. Soc. London Ser. B*, **369**, 20130315. 10.1098/rstb.2013.0315 PMC4052857 24914148

[bb32] Mendez, D., Watkins, H., Qiao, S., Raines, K. S., Lane, T. J., Schenk, G., Nelson, G., Subramanian, G., Tono, K., Joti, Y., Yabashi, M., Ratner, D. & Doniach, S. (2016). *IUCrJ*, **3**, 420–429. 10.1107/S2052252516013956 PMC5094444 27840681

[bb33] Pande, K., Donatelli, J. J., Malmerberg, E., Foucar, L., Bostedt, C., Schlichting, I. & Zwart, P. H. (2018). *Proc. Nat. Acad. Sci. USA*, **115**, 11772–11777. 10.1073/pnas.1812064115 PMC6243272 30373827

[bb34] Pedrini, B., Menzel, A., Guizar-Sicairos, M., Guzenko, V. A., Gorelick, S., David, C., Patterson, B. D. & Abela, R. (2013). *Nat. Commun.* **4**, 1647. 10.1038/ncomms2622 23552062

[bb35] Poon, H. C. & Saldin, D. K. (2011). *Ultramicroscopy*, **111**, 798–806. 10.1016/j.ultramic.2010.11.003 21168272

[bb36] Poulos, A. S., Constantin, D., Davidson, P., Pansu, B., Freyssingeas, É., Madsen, A. & Chanéac, C. (2010). *J. Chem. Phys.* **132**, 091101. 10.1063/1.3330920 20210381

[bb37] Saldin, D. K., Poon, H. C., Schwander, P., Uddin, M. & Schmidt, M. (2011). *Opt. Express*, **19**, 17318–17335. 10.1364/OE.19.017318 21935096

[bb38] Saldin, D. K., Shneerson, V. L., Fung, R. & Ourmazd, A. (2009). *J. Phys. Condens. Matter*, **21**, 134014. 10.1088/0953-8984/21/13/134014 21817489

[bb39] Schroer, M. A., Gutt, C., Lehmkühler, F., Fischer, B., Steinke, I., Westermeier, F., Sprung, M. & Grübel, G. (2015). *Soft Matter*, **11**, 5465–5472. 10.1039/c5sm00609k 26061482

[bb40] Schroer, M. A., Westermeier, F., Lehmkühler, F., Conrad, H., Schavkan, A., Zozulya, A. V., Fischer, B., Roseker, W., Sprung, M., Gutt, C. & Grübel, G. (2016). *J. Chem. Phys.* **144**, 084903. 10.1063/1.4941563 26931722

[bb41] Starodub, D., Aquila, A., Bajt, S., Barthelmess, M., Barty, A., Bostedt, C., Bozek, J. D., Coppola, N., Doak, R. B., Epp, S. W., Erk, B., Foucar, L., Gumprecht, L., Hampton, C. Y., Hartmann, A., Hartmann, R., Holl, P., Kassemeyer, S., Kimmel, N., Laksmono, H., Liang, M., Loh, N. D., Lomb, L., Martin, A. V., Nass, K., Reich, C., Rolles, D., Rudek, B., Rudenko, A., Schulz, J., Shoeman, R. L., Sierra, R. G., Soltau, H., Steinbrener, J., Stellato, F., Stern, S., Weidenspointner, G., Frank, M., Ullrich, J., Strüder, L., Schlichting, I., Chapman, H. N., Spence, J. C. H. & Bogan, M. J. (2012). *Nat. Commun.* **3**, 1276. 10.1038/ncomms2288 23232406

[bb42] Treacy, M. M. J. & Borisenko, K. B. (2012). *Science*, **335**, 950–953. 10.1126/science.1214780 22363003

[bb43] Treacy, M. M. J., Gibson, J. M., Fan, L., Paterson, D. J. & McNulty, I. (2005). *Rep. Prog. Phys.* **68**, 2899–2944.

[bb44] Treacy, M. M. J., Kumar, D., Rougée, A., Zhao, G., Buseck, P., McNulty, I., Fan, L., Rau, C. & Gibson, J. M. (2007). *J. Phys. Condens. Matter*, **19**, 455201.

[bb45] Warren, B. E. (1990). *X-ray diffraction.* New York: Dover Publications.

[bb46] Wochner, P., Gutt, C., Autenrieth, T., Demmer, T., Bugaev, V., Ortiz, A. D., Duri, A., Zontone, F., Grübel, G. & Dosch, H. (2009). *Proc. Nat. Acad. Sci. USA*, **106**, 11511–11514. 10.1073/pnas.0905337106 PMC2703671 20716512

[bb47] Zaluzhnyy, I. A., Kurta, R. P., André, A., Gorobtsov, O. Y., Rose, M., Skopintsev, P., Besedin, I., Zozulya, A. V., Sprung, M., Schreiber, F., Vartanyants, I. A. & Scheele, M. (2017*a*). *Nano Lett.* **17**, 3511–3517. 10.1021/acs.nanolett.7b00584 28485967

[bb48] Zaluzhnyy, I. A., Kurta, R. P., Mukharamova, N., Kim, Y. Y., Khubbutdinov, R. M., Dzhigaev, D., Lebedev, V. V., Pikina, E. S., Kats, E. I., Clark, N. A., Sprung, M., Ostrovskii, B. I. & Vartanyants, I. A. (2018). *Phys. Rev. E*, **98**, 052703.

[bb49] Zaluzhnyy, I. A., Kurta, R. P., Sulyanova, E. A., Gorobtsov, O. Y., Shabalin, A. G., Zozulya, A. V., Menushenkov, A. P., Sprung, M., Krówczyński, A., Górecka, E., Ostrovskii, B. I. & Vartanyants, I. A. (2017*b*). *Soft Matter*, **13**, 3240–3252. 10.1039/c7sm00343a 28402369

[bb50] Zaluzhnyy, I. A., Kurta, R. P., Sulyanova, E. A., Gorobtsov, O. Y., Shabalin, A. G., Zozulya, A. V., Menushenkov, A. P., Sprung, M., Ostrovskii, B. I. & Vartanyants, I. A. (2015). *Phys. Rev. E*, **91**, 042506. 10.1103/PhysRevE.91.042506 25974515

